# The Relationship between Emotional Intelligence and Job Stress among Nurses in Shiraz, Iran

**DOI:** 10.21315/mjms2018.25.6.10

**Published:** 2018-12-28

**Authors:** Tayebeh Rakhshani, Zahra Motlagh, Vahid Beigi, Marzieh Rahimkhanli, Mostafa Rashki

**Affiliations:** 1 Research Center for Health Sciences, Institute of Health, Shiraz University of Medical Sciences, Shiraz, Iran; 2 Ophthalmology Research Center, Department of Ophthalmology, School of Medicine, Shiraz University of Medical Sciences, Shiraz, Iran; 3 Mashad University of Medical Sciences, Mashad, Iran

**Keywords:** emotional intelligence, job stress, nurses, cross sectional study

## Abstract

**Background:**

Nursing is a stressful occupation, and nurses face multiple stressors daily. Emotional intelligence causes an individual to overcome environmental stresses. The aim of the present study was to determine the relationship between emotional intelligence and job stress among nurses in Shiraz.

**Methods:**

A cross-sectional study was conducted with 500 nurses, selected through multistage cluster sampling, from the hospitals in Shiraz in 2017. The data collection tools were the Siberia Schering’s Emotional Intelligence Standard Questionnaire and the Hospital Job Stress Standard Questionnaire, completed through the self-report method. The data were analysed using SPSS 22 software.

**Results:**

The mean scores of emotional intelligence and job stress were 113.59 ± 14.70 (total score = 165) and 97.10 ± 14.27 (total score = 175), respectively. The correlation test showed that there was an inverse relationship between emotional intelligence and job stress (*r* = −0.474, *P* < 0.001). Also, the multiple linear regression analysis showed that self-awareness, social consciousness, and income predicted 25% of the job stress in the subjects under study (*r*^2^ = 0.25).

**Conclusion:**

Regarding the relatively strong and inverse relationship between the nurses’ emotional intelligence and job stress, it is suggested that emotional intelligence workshops be included in the in-service training programs of the nurses.

## Introduction

Nursing is a stressful occupation and nurses face several stressors in their work environment daily and continuously, including overwork, individual conflicts, shift work, dealing with deaths, lack of mental support, conflicts with doctors, ambiguity in the level of authority, and they experience a high level of stress every day (1–5). Hence, the National Association of Safety Professionals in the United States has introduced nursing at the top of 40 professions with a high incidence of stress-related illnesses and believes that nursing is likely to be at the forefront of stressful healthcare occupations (6).

One of the ways to cope with job stress is emotional intelligence, which is now being considered by intellectuals more than ever. Research has shown that under the best conditions, mental intelligence determines only 20% of a person’s success, and the remaining 80% depends on other factors like emotional intelligence (6). Emotional intelligence refers to a set of non-cognitive abilities, efficiencies, and skills that influence an individual’s ability to succeed in coping with environmental pressures and assists him/her to overcome environmental stresses and needs (7, 8). According to Delpasand, the components of emotional intelligence are self-awareness, self-control, self-motivation, empathy or social consciousness, and social skills (9).

Since nurses are physically and emotionally exposed to different working conditions compared to other occupations, they have to increase their emotional skills to adapt themselves to the abnormal conditions of the work environment (10). If nurses do not have adequate skills to control their emotions, they will not be able to maintain their peace of mind when communicating with patients, especially in different crises situations (11). Those who use their emotional intelligence are more compatible with their surroundings and have greater self-confidence and awareness of their abilities (12).

Rostami et al. showed a relationship between emotional intelligence and different ways of coping with stress in the nurses of the hospitals affiliated to Kermanshah University of Medical Sciences; emotional intelligence could lead to the use of effective countermeasures against stress (problem-centred) (13). In a study by Kheirmand et al. aimed at determining the relationship between emotional intelligence and nurses’ job stress, it was found that increased emotional intelligence led to a decrease in the nurses’ job stress (6). A study by Nooryan et al. showed that nurses and doctors experienced a lot of stress and anxiety, and the effectiveness of teaching emotional intelligence components and sharing information about emotional intelligence at the workplace could have a remarkable impact on adapting to job stress or anxiety (14). The achievement of the highest level of productivity is one of the factors affecting human resources, but the job stress of the nurses does not allow the achievement of high levels of productivity. Considering the importance of emotional intelligence in reducing job stress, this study was conducted to determine the relationship between emotional intelligence and job stress among the nurses in Shiraz. It was carried out to understand the nurses’ emotional intelligence and identify the stressors, limit them or raise the nurses’ level of scientific and professional knowledge to increase their compatibility with different situations and provide a convenient environment for them to work.

## Materials and Methods

### Study Design and Participants

A cross-sectional study was conducted in Shiraz in 2017. The study population consisted of the nurses working in public and private hospitals in Shiraz. The inclusion criteria were at least one year of work experience and satisfaction with participation in the study.

According to the highest standard deviation obtained from the aspects of emotional intelligence in a similar study (SD = 0.52) (6) and considering the confidence level of 95% and *d* = 0.05, the estimated sample size was 412, but due to the likely incomplete completion of the questionnaire and for more confidence, a sample size of 500 nurses was considered.

The sampling was done through a multistage cluster method, where public or private hospitals were considered as clusters; five public hospitals as well as five private ones were selected randomly. Then, 50 nurses were selected from each hospital through simple random sampling.

### Instruments

The instruments used in this study were the Siberia Schering’s Emotional Intelligence Standard Questionnaire, the Hospital Job Stress Standard Questionnaire (HSS), and demographic characteristics.

The Siberia Schering’s Emotional Intelligence Standard Questionnaire contained 33 questions with five sub-scales of self-awareness, self-regulation, motivation, empathy, and social skills, which were scored with a 5-point Likert scale as always = 5, often = 4, sometimes =3, rarely = 2 and never =1. Some of the questions were scored in a reverse order. The scores of the questionnaire ranged from 33 to 165, and the highest score indicated the highest emotional intelligence (9). The scores ranging from 33–65 showed low emotional intelligence, 66–98 showed moderate, 99–131 showed good, and 132–165 showed excellent emotional intelligence (15). The questionnaire was reviewed by Kheirmand in 2015 on the nurses of Alzahra Hospital in Isfahan, and its reliability was reported to be 0.86 using Cronbach’s alpha (6).

The HSS consisted of 35 questions with seven sub-scales, such as demand, control, support of authorities, support of colleagues, communication, role, and changes. Scoring the sub-scales of the questionnaire in the 5-option Likert scale ranged from never = 5 to always = 1. Scoring the sub-scales of demand and relation was done reversely (from always = 5 to never = 1). The lowest score was 35, and the highest was 175. Higher scores indicated higher levels of stress experienced by the individuals. The scores 35–69 showed low stress, 70–104 showed moderate stress, and 105–175 indicated severe stress (16). The validity of this questionnaire was verified in an extensive reading by Marzabadi and Fesharaki, and its reliability was calculated to be 0.78 using Cronbach’s alpha (17).

The demographic information questionnaire included age, gender, marital status, number of children, education level, nursing experience, rank, salary, and work department.

### Data Collection Method

To collect the data, the researchers first got permission from the head nurse of each hospital to visit the subjects in-person. After introducing themselves and expressing the objectives, process, and importance of the study and ensuring that the subjects’ information would remain confidential and the results of the research would be generally used as a research plan, they delivered the job stress, emotional intelligence and demographic questionnaires to the subjects. Because of the workload of the nurses and the lack of accountability in receiving the questionnaire, the researchers decided to collect the questionnaires the next day.

### Data Analysis

The data were entered into the SPSS 22 software and analysed using the Independent *t*-test, one-way ANOVA, Pearson correlation, and simple and multiple linear regression. In the multiple linear regression, job stress was entered as dependent variable (the total score of all sub-scales in job stress was considered as one single score for job stress). All sub-scales of emotional intelligence and the demographic characteristics were considered as independent and confounding variables, respectively.

## Results

Of all the subjects, 79.4% (397 people) were female and 18% (90 people) were male. Most of the subjects were married (58%, 290 people) and had a bachelor’s degree (84%, 420 people). It was found that 64.4% (322 people) had < 10 years of work experience and 80.6% (403 people) had an income of < 500 US Dollar (USD). Most of the individuals (35.2%, 176 people) were employed on a contractual basis, and most of them (86.4%, 432 people) were working as ordinary nursing staff. The demographic characteristics of the study participants are presented in Table 1. Possible range, observed range, mean and standard deviation of emotional intelligence, and job stress as well as their dimensions are shown in Table 2. The mean and standard deviation of emotional intelligence was 113.59 ± 14.70 and that of job stress was 97.10 ± 14.27 (Table 2).

The mean and standard deviation of emotional intelligence and job stress according to demographic variables are shown in Table 3.

The one-way analysis of variance (ANOVA) showed a significant relationship between emotional intelligence and type of employment (*P* = 0.033) and marital status (*P* = 0.002). There was no significant relationship between emotional intelligence and gender, rotating working shift, education level, service record, income, and job position (*P* > 0.05) (Table 3). There was a significant relationship between job stress and type of employment (*P* = 0.019) and income (*P* = 0.005). However, no significant relationship was found between job stress and gender, rotating working shift, education level, service record, job position, and marital status (*P* > 0.05) (Table 3).

The correlation test showed that there was an inverse relationship between emotional intelligence and job stress (*r* = −0.474, *P* < 0.001). The correlation coefficient between emotional intelligence and job stress sub-scales are presented in Table 4. The multiple linear regression showed that self-awareness (β = −1.007, *P* < 0.001), social consciousness (β = −0.76, *P* < 0.003), and income (β = −6.08, *P* < 0.001) were the significant predictors of job stress, accounting for 25% job stress changes (*r*^2^ = 0.25) (Table 5).

## Discussion

The aim of this study was to determine the relationship between emotional intelligence and job stress among nurses of the hospitals in Shiraz. The results of this study showed that there was a relatively strong and inverse relationship between the nurses’ emotional intelligence and their job stress. According to the studies by Kherandish et al. (18) on the nurses working in Labafinejad Hospital in Tehran, Samaei et al. (19) on the nurses in Kerman hospitals, Karimi et al. (20) on Australian nurses, and Hong et al. (21) on South Korean nurses, increased emotional intelligence led to decreased job stress in the nurses, which is consistent with the results of the present study. In Görgens-Ekermans’s study on South African nurses, emotional control and emotional management were inversely associated with self-reported stress (22). In the study by Hamdy El-Sayed et al. on the faculty members working in Faculty of Nursing in Zagazig University, and those by Mansour et al. and Baezzat et al. on university employees, increased emotional intelligence led to decreased job stress, which is consistent with the results of the present study (23–25). The findings indicated that individuals with high emotional intelligence had self-awareness, self-control, self-motivation, empathy, and social skills. They were more capable of controlling and managing stress when confronted with stressful situations because of logical and rational analysis of the situations and problems, tolerance and adaptation to stressful situations, past experiences, and hope of improving the situation (26).

In this study, the mean score of emotional intelligence was 113.59 ± 14.70. The mean score of emotional intelligence in the study by Rezaie et al. (27) on the nurses of Bushehr Main Hospital of Medical Sciences was 100.23 ± 6.15, while it was 112.03 ± 9.04 in the study by Delpasand (9) on the nurses of Tehran Social Security Hospital, and 110.20 ± 7.83 in Tourani’s study (15) on the nurses in the emergency departments of the educational-medical centres of Iran University of Medical Sciences. Thus, all the results are consistent with the present study.

In the present study, the mean score of the nurses’ job stress was 97.10 ± 14.27, indicating that the nurses experienced moderate levels of stress. According to the study of Azadi et al. (28), the mean score of job stress of the nurses in the hospitals of Isfahan was 102.08 ± 17.64, and it was 104.11 ± 16.42 in the study by Adib-Hajbaghery et al. (29) on the nurses in the hospitals of Shahid Beheshti University of Medical Sciences. It is consistent with the findings of the present study, indicating that nurses generally experience moderate levels of stress.

In the study by Nasiry Zarrin Ghabaee et al. (30) on the nurses in Sari hospitals, the mean score of job stress was 115.02 ± 20.94, which indicated a high level of stress and is not consistent with the present study. Considering the fact that in Nasiry Zarrin Ghabaee’s study, many nurses were simultaneously working in other departments like the psychiatric department, and most of the studies carried out in this field have mentioned high workload as one of the major stressors (30), the higher job stress of the nurses in Nasiry Zarrin Ghabaee’s study, compared to the present one, could be justified.

In the present study, most nurses (65.7%) experienced moderate levels of stress. In the studies by Masoumy et al. (16) and Rahmani et al. (31), a majority of nurses (46.4% and 49.2%, respectively) had severe stress, which is not consistent with the present study. This difference may be attributed to the type of the target group in the studies. In this study, the nurses working in all sections were studied while in the studies by Masoumy et al. and Rahmani et al. the nurses working in special sections were evaluated. The nurses working in special sections might suffer from the highest level of stress due to the special conditions caused by the work environment and the patients. Among the stressors of the special sections are the high workloads, the need for prompt and timely response to urgent situations, the heavy responsibility of patient care, working relations with other nurses and health staff, communication and conversation with patients and their relatives, and high levels of knowledge and skills required to work in the section.

In the present study, permanent nurses obtained significantly lower scores of stress than conscripted ones, and the nurses with higher incomes obtained lower scores of stress compared to those with lower incomes. In the study by Hamdy El-Sayed et al. (23), a significant inverse correlation was found between job stress and service record and income, which is consistent with the present study. Since employment means working permanently and having a higher income, it is among the social factors affecting health and contributes to better social welfare, reduced stress, and improvement of individuals’ mental health. In a study conducted on managers in Iran in 2013, a significant relationship was found between the emotional intelligence of men and women, which was not consistent with the results of our study (32).

One of the strengths of this study is the large sample size (500 people). Another strength is the random cluster sampling from 10 hospitals, and both strengths can somewhat enhance the generalisability of the results.

One weakness of this study is that it is cross-sectional. Therefore, in order to confirm the results, it is better to design and conduct interventional studies on the effect of emotional intelligence on nurses’ job stress.

## Conclusion

The findings of this study, based on the mean score of job stress and emotional intelligence of nurses, showed that the job stress and emotional intelligence levels of nurses working in the hospital was moderate and good, respectively, and there was a relationship between job stress and emotional intelligence. Therefore, educational classes based on the components of emotional intelligence can be effective in reducing stress in nursing. In different nurse communities, emotional intelligence interventions should be designed and performed based on evidence-based studies. Considering that income was predictive of job stress among the demographic variables, the proposal to increase the revenues of the managers and policymakers in the healthcare sector could be effective in reducing job stress and increasing the efficiency, and quality of healthcare services. It is also suggested that topics related to the concept of emotional intelligence, such as emotion management, social consciousness, self-awareness, etc., should be included in the academic curriculum of nursing students, and from the beginning, nursing students should be familiar with the concept of emotional intelligence and its dimensions.

## Figures and Tables

**Table 1:** Demographic characteristics of the participants in the study

Variables	Groups	*N*	%
**Gender**	Female	397	79.4
	Male	90	18.0
	Unspecific (no response)	13	2.6
**Marital status**	Single	194	38.8
	Married	290	58.0
	Divorced	4	0.8
	Widow	2	0.4
	Unspecific (no response)	10	2.0
**Service record**	< 10 years	322	64.4
	10–20 years	124	24.8
	> 20 years	24	4.8
	Unspecific (no response)	30	6.0
**Income, USD**	< 500	403	80.6
	500–700	64	12.8
	> 700	6	1.2
	Unspecific (no response)	27	5.4
**Education level**	Diploma	28	5.6
	Associate degree	21	4.2
	Bachelor’s degree	420	84
	Master’s degree	21	4.2
	Unspecific (no response)	10	2.0
**Employment type**	Conscripted	166	33.2
	Contractual	176	35.2
	Temporary	66	13.2
	Permanent	69	13.8
	Unspecific (no response)	23	4.6
**Job position**	Ordinary staff	432	86.4
	Head nurse	48	9.6
	Supervisor	13	1.4
	Unspecific (no response)		4.0
**Rotating working shift**	Yes	440	88.0
	No	40	8.0
	Unspecific (no response)	20	4.0

**Table 2:** Possible range, observed range, mean and standard deviation of emotional intelligence and job stress

Variable	Dimension	Possible range	Observed range	mean ± SD	Median
Emotional intelligence	Self-awareness	8–40	12–40	29.01 ± 4.70	29
	Self-control	7–35	11–33	22.98 ± 4.16	23
	Self-motivation	7–35	13–31	23.08 ± 33.25	23
	Social consciousness	6–30	10–30	20.62 ± 3.62	21
	Social skills	5–25	8–24	17.04 ± 3.14	17

Total score		33–165	65–148	113.59 ± 14.70	115

Job stress	Demand	8–40	10–40	24.27 ± 5.36	24
	Relation	4–20	4–20	11.10 ± 3.31	11
	Control	6–30	6–28	16.51 ± 3.79	17
	Support of colleagues	4–20	4–20	10.98 ± 3.92	11
	Support of authorities	5–25	5–25	14.16 ± 3.52	14
	Role	5–25	5–23	11.78 ± 3.98	12
	Changes	3–15	3–15	8.34 ± 2.30	8

Total score		35–175	53–135	97.10 ± 14.27	98

**Table 3:** Mean and standard deviation of emotional intelligence and job stress related to the demographic variables

Variable		Emotional intelligence		Job stress	
	
		Mean ± SD	*P-*value	Mean ± SD	*P*-value
Gender	Female	114.22 ± 14.67	0.054*	97.09 ± 14.51	0.748*
	Male	110.42 ± 14.79		97.64 ± 13.03	
Marital status	Single	110.39 ± 15.31	0.002**	98.40 ± 15.11	0.598**
	Married	115.73 ± 13.99		96.14 ± 13.78	
	Divorced	114 ± 9.89		99	
	Widow	121 ± 17.67		103.50 ± 9.19	
Education level	Diploma	114.17 ± 14.07	0.444**	97.21 ± 12.17	0.912**
	Associate degree	108.13 ± 17.68		99.76 ± 17.80	
	Bachelor’s degree	113.67 ± 14.63		97.08 ± 14.19	
	Master’s degree	116.22 ± 14.44		95.50 ± 15.64	
Employment type	conscripted	110.70 ± 13.59	0.033**	99.83 ± 13.73	0.019**
	contractual	110.62 ± 15.54		95.90 ± 14.27	
	temporary	114.88 ± 14.39		97.12 ± 12.30	
	permanent	115.25 ± 14.48		92.19 ± 16.33	
Income, USD	< 500	113.64 ± 14.37	0.115**	98.22 ± 13.99	0.005**
	500–700	115.92 ± 15.38		91.12 ± 14.09	
	> 700	98.66 ± 22.54		87.75 ± 17.67	
Service record	Fewer than 10 years	113.20 ± 13.83	0.792**	98.17 ± 1348	0.081**
	10 to 20 years	114.39 ± 16.48		96.91 ± 14.89	
	Over 20 years	113.72 ± 16.83		91.05 ± 16.66	
Job position	Ordinary staff	113.19 ± 14.53	0.295**	97.43 ± 13.97	0.295**
	Head nurse	116.62 ± 16.10		94 ± 17.14	
	Supervisor	110 ± 12.34		102 ± 13.11	
Rotating working shift	Yes	113.36 ± 14.37	0.093*	97.42 ± 14.05	0.204*
	No	118 ± 15.02		93.52 ± 16.96	

* Independent *t*-test

** One way ANOVA

**Table 4:** Correlation coefficient matrix between emotional intelligence and job stress sub-scales

	1	2	3	4	5	6	7	8	9	10	11	12
1. Self-awareness	1											
2. Self-control	0.508**	1										
3. Self-motivation	0.504**	0.542**	1									
4. Social consciousness	0.542**	0.543**	0.473**	1								
5. Social skills	0.466**	0.409**	0.337**	0.474**	1							
6. Demand	−0.052	−0.140**	−0.118*	−0.085	−0.090	1						
7. Relation	−0.208**	−0.208**	−0.175**	−0.177**	−0.126**	0.598**	1					
8. Control	−0.319**	−0.266**	−0.240**	−0.269**	−0.228**	−0.136**	−0.043	1				
9. Support of colleagues	−0.252**	−0.215**	−0.199**	−0.224**	−0.180**	−0.164**	−0.020	0.496**	1			
10. Support of authorities	−0.185**	−0.150**	−0.129**	−0.172**	−0.171**	−0.181**	−0.082	0.551**	0.707**	1		
11. Role	−0.454**	−0.278**	−0.341**	−0.380**	−0.307**	−0.162**	0.059	0.479**	0.420**	0.370**	1	
12. Changes	−0.209**	−0.159**	−0.207**	−0.218**	−0.143**	−0.153**	0.003	0.515**	0.623**	0.660**	0.324**	1

* *P*-value < 0/05

** *P*-value < 0/01

**Table 5:** Factors associated with job stress

	Simple linear regression				Multiple linear regression			
	
	Predictor variables	Unstandardised β	*P*	*r*^2^	Predictor variables	Unstandardised β	*P*	*r*^2^
Job stress	Self-control	−1.31	< 0.001	0.147	Self–awareness	−1.007	< 0.001	0.257
	Self-awareness	−1.29	< 0.001	0.19	Income	−6.08	0.001	
	Self-motivation	−1.60	< 0.001	0.12	Social consciousness	−0.76	0.003	
	Social consciousness	−1.50	< 0.001	0.13				
	Social skills	−1.49	< 0.001	0.10				
	Gender	−0.55	0.80	0.00				
	Marital status	−1.81	0.271	0.004				
	Education level	−0.73	0.644	0.001				
	Employment type	−2.17	0.007	0.025				
	Income	−6.52	0.002	0.034				
	Service record	−2.52	0.067	0.012				
	Job position	−1.72	0.461	0.002				
	Rotating working shift	−3.90	0.250	0.005				
